# Stereotactic Radiotherapy for Locally Recurrent and Metastatic Soft Tissue Sarcoma

**DOI:** 10.7759/cureus.84886

**Published:** 2025-05-27

**Authors:** Anastasia Stergioula, Stefania Kokkali, Theodoros Kormas, Nikolaos Memos, Evaggelos Pantelis

**Affiliations:** 1 Department of Radiotherapy, Iatropolis Clinic, Athens, GRC; 2 Oncology Unit, Hippocratio General Hospital, Athens, GRC; 3 Department of Orthopedic Surgery, Agios Savvas Anticancer Hospital, Athens, GRC; 4 Department of Surgery, Aretaieion Hospital, Athens, GRC; 5 School of Medicine, National and Kapodistrian University of Athens, Athens, GRC

**Keywords:** cyberknife, helical tomotherapy, sbrt, soft tissue sarcoma, stereotactic radiotherapy

## Abstract

Background

Approximately half of soft tissue sarcoma (STS) patients experience local relapse or develop distant metastases during the course of the disease. The radioresistance of the majority of STS tumors necessitates increased biologically effective doses (BEDs) to achieve favorable outcomes. These doses can be safely delivered using stereotactic radiotherapy (SRT) techniques.

Methods

The outcome of 36 locally and distantly recurrent STS lesions in 16 patients treated with SRT was reviewed. A variety of STS histologies and locations were included, involving the lungs, abdomen, extremities, trunk, spine, and brain. A median marginal BED of 96 Gy₁₀ (range: 48-151 Gy₁₀) was delivered, calculated using an α/β ratio of 10 Gy. Kaplan-Meier analysis was performed to assess the local recurrence-free survival on a per-target basis.

Results

The median follow-up was 13 months (range: three to 27 months). Actuarial local control rates of 26/26 (100%) and 15/17 (88%) were observed at 12 and 18 months post-SRT, respectively. SRT was associated with minimal toxicities, except for one patient who presented with late skin toxicity of the upper extremity.

Conclusions

Results of this study indicate that SRT should be considered an effective treatment option for the management of selected locally recurrent and metastatic STS patients.

## Introduction

Soft tissue sarcoma (STS) is a heterogeneous group of rare tumors that originate from mesenchymal tissue [1]. Standard treatment for localized primary STS involves a multimodal approach, incorporating surgery and radiotherapy (RT) with or without chemotherapy (CHT) [2,3]. This approach provides long-term local control (LC) and an overall survival (OS) rate of 81% at five years [4]. Approximately half of STS patients relapse locally or develop distant metastases at some point during the disease course [5,6]. In these settings, management primarily relies on systemic therapy, including CHT, targeted agents, and immunotherapy [7,8]. However, overall response rates for systemic therapy are relatively poor, with nearly all patients eventually progressing on therapy [9]. Surgery is often used for achieving LC, but not all patients are suitable candidates for surgical resection due to the potential high risk of complications, which may be exacerbated by factors such as age, comorbidities, and prior therapies [10,11]. RT is used for treating unresectable recurrent tumors or for alleviating symptoms in advanced stages of the disease. Given the relative radioresistance of sarcomas, conventionally fractionated RT may be inadequate to provide durable tumor control or even effective palliation.

Stereotactic RT (SRT) delivers a high biologically effective dose (BED) to a well-defined target in a small number of fractions. Although few studies, mostly retrospective, on SRT in STS are available in the literature, the data demonstrate excellent LC and highlight the survival benefit associated with locally aggressive treatment [12,13]. Thus, SRT may be a favorable treatment option for selected STS patients to provide effective LC and symptom relief with limited toxicity.

This study presents an institutional experience evaluating the clinical outcomes of patients with locally recurrent or metastatic STS treated with SRT techniques.

## Materials and methods

Patient population

STS patients treated in our clinic from 2020 to 2024 using SRT were included in this retrospective study. The inclusion criteria targeted patients classified as “oligometastatic” or in an “oligoprogressive state” [13], who had shown disease progression after or under systemic therapies, as well as those with local recurrence. Indications for SRT had been discussed by an experienced multidisciplinary sarcoma board for all patients. Retrieved patient data included demographic information and clinical details involving tumor histology, treatment site, tumor size, and type of any previous treatments. Information regarding the prescribed dose at the periphery of each target and the number of fractions was extracted from each patient’s treatment plan. Dose and fractionation protocols were tailored to each case. Therefore, in addition to the delivered physical doses, the BED for each target was calculated using the linear quadratic model and an α/β ratio of 10 Gy.

SRT protocols

SRT treatments were delivered using the CyberKnife™ and the Helical TomoTherapy™ image-guided RT platforms (Accuray Inc., Sunnyvale, CA, USA) [14,15]. All intracranial, spinal, and thorax lesions were treated with the CyberKnife system due to its advanced real-time intrafraction target motion management systems. All other lesions were treated with the TomoTherapy platform, which allows acquisition of CT images of the patient at treatment position and offline adaptation of the delivered RT dose distributions. These CT images were obtained prior to each fraction to ensure accurate patient registration at the treatment position and reduce setup uncertainties. Immobilization devices (e.g., vacuum bags) were used to minimize intrafraction patient movements. Additionally, when TomoTherapy dose delivery exceeded five minutes, intrafraction imaging was employed by splitting each treatment fraction into two complete parts, with a second CT scan acquired before delivering the second part.

Treatment planning was based on non-contrast-enhanced CT images of the patient’s treated anatomy. The gross tumor volume (GTV) was identified on the planning CT images with the aid of corresponding contrast-enhanced T1-weighted and T2-weighted MRI and, if available, PET/CT data. For lung lesions, a four-dimensional CT (4DCT) scan was performed, enabling the delineation of the GTV across the different respiratory phases. A lung-optimized simulation study was performed prior to treating each lung tumor to assess the suitability of the Synchrony-Lung Optimized Treatment (Synchrony-LOT™) option for delivery [14]. Tumors clearly visible in both live X-ray images were treated using the 2-view Synchrony-LOT option. For tumors visible in only one live x-ray image, the 1-view Synchrony-LOT was used, while tumors not visible in either x-ray image were treated using the 0-view Synchrony-LOT option. In 1-view cases, the internal target volume (ITV) was delineated based on the GTVs contoured at the expiration and inspiration phases, ensuring coverage of untracked tumor motion using the 4DCT data. In 0-view cases, the ITV was defined based on the maximum intensity projection CT volume derived from the patient’s 4DCT scan. Spinal lesions were delineated according to the recommendations of the International Spine Radiosurgery Consortium [16]. The planning target volume (PTV) was defined by applying an isotropic expansion of the GTV or ITV by 1 mm in the intracranial, 2 mm in the spinal, and 5 mm in the rest of the treated lesions. The dose prescription was performed using a risk-adaptive dose fractionation approach considering target location and patient morbidity.

Assessment of outcome and toxicity

LC was assessed for each lesion separately, comparing pretreatment and posttreatment radiologic imaging data. Progression was defined as a ≥20% increase in the sum of diameters of target lesions, according to Response Evaluation Criteria in Solid Tumors (RECIST) version 1.1. Radiation toxicity grading was assessed using the Common Terminology Criteria for Adverse Events version 5.0.

Statistical methods

Kaplan-Meier analysis was used to determine the time to event, which involved tumor progression or death. Local recurrence-free survival (LRFS) was analyzed on a per-lesion basis and defined as the time from the SRT to progression of the treated lesion or censored at last follow-up. All statistical analyses were performed using RStudio: Integrated Development for R (PBC, Boston, MA, USA).

## Results

Clinical and treatment details of the studied population

The studied cohort comprised 16 patients with a median age of 65 years (range: 33-87 years) at the time of SRT. In half of the patients, the primary disease was located in the extremity and superficial trunk, and in the retroperitoneum, thoracic, or abdominal viscera in the rest of the patients. The most common histologic types were undifferentiated pleomorphic sarcoma (5/16, 31%) and leiomyosarcoma (5/16, 31%), with less common histologies including angiosarcoma, liposarcoma, and malignant peripheral nerve sheath tumors. A total of 36 lesions were treated using SRT (Table 1). The majority of the lesions were located in the lungs (19/36, 53%), with the rest distributed in the abdomen (6/36, 17%), extremities (5/36, 14%), trunk (3/36, 8%), spine (2/36, 6%), and brain (1/36, 3%). Three out of the five relapses in the extremities had had preoperative RT, receiving a dose of 50 Gy in 25 fractions. None of the remaining lesions had had any prior RT. The median size of the lesions at the time of SRT was 2 cm (range: 1-8 cm). The median PTV volume was 13 cm³ (range: 2-285 cm³). A median dose of 30 Gy (range: 24-60 Gy) was prescribed and delivered in 1 to 13 fractions (median: 3 fractions). The median BED for the treated lesions was 96 Gy₁₀ (range: 48-151 Gy₁₀).

**Table 1:** Clinical and treatment details of the lesions treated with SRT ^a^ Frequency (%) or median (min, max) BED, biologically effective dose; SRT, stereotactic radiotherapy

Characteristic	N = 36^a^
Location	
Lungs	19 (53%)
Abdomen	6 (17%)
Extremities	5 (14%)
Trunk	3 (8%)
Spine	2 (6%)
Brain	1 (3%)
Size (cm)	2 (1, 8)
Volume (cm³)	13 (2, 285)
Prescribed dose (Gy)	30 (24, 60)
Number of fractions	3 (1, 13)
BED (Gy₁₀)	96 (48, 151)

Outcomes

**Figure 1:**
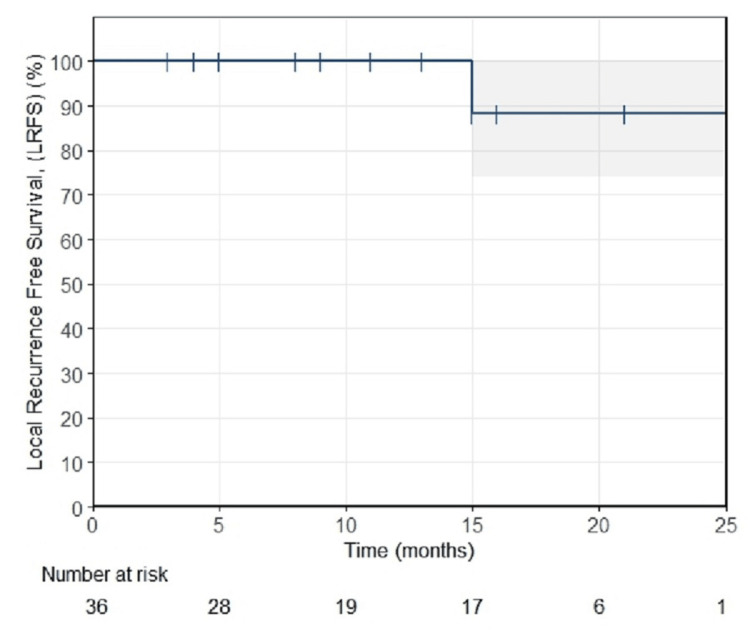
Kaplan-Meier LRFS data of the studied patient cohort LRFS, local recurrence-free survival

The median follow-up of the studied patient cohort was 13 months (range: three to 27 months). Kaplan-Meier analysis of LRFS for the treated lesions is shown in Figure 1, demonstrating actuarial LC rates of 26/26 (100%) and 15/17 (88%) at 12 and 18 months post-SRT, respectively. At the time of analysis, all patients were alive, with or without evidence of disease, except for one who died five months post-SRT due to surgical complications following the resection of the lung metastasis. The OS rate was found equal to 9/10 (90%) at 18 months.

Toxicity

SRT treatments were well tolerated, with no patients having developed grade ≥2 acute gastrointestinal or genitourinary toxicities. None of the patients treated for lung metastases experienced symptomatic pneumonitis. However, radiographic findings indicative of asymptomatic pneumonitis were revealed in the lung tissue surrounding one of the treated lesions. Furthermore, none of the patients treated for lesions in the vicinity of the chest wall developed rib fractures. Acute skin toxicity (grade 2) was observed in a single patient treated for local recurrence in the upper extremity with a dose of 57.6 Gy in 12 consecutive daily fractions. Six months post-SRT, the patient displayed severe late skin toxicity (grade 3) involving radiation necrosis and infection, which was managed with antibiotics.

## Discussion

The use of SRT in the management of locally recurrent and metastatic STS was evaluated. Sixteen patients presented with a total of 36 lesions of various histologies and were included in this retrospective study. All patients had been initially treated with CHT with or without surgery and, upon disease progression, were referred for SRT. A median marginal BED of 96 Gy₁₀ was delivered, achieving LC rates of 26/26 (100%) and 15/17 (88%) at 12 and 18 months post-SRT, respectively. An OS rate of 90% at 18 months post-SRT was calculated. All SRT treatments were well tolerated, with no patients experiencing grade ≥2 acute or late toxicities, except for one case of severe grade 3 skin toxicity.

Table 2 compares the results of this study with corresponding data reported in the literature. Overall, the presented data suggest that BED values greater than 95 Gy₁₀ are typically delivered to metastatic STS lesions located in the lung, liver, lymph nodes, or other soft tissue sites [17,18]. These BED values have been associated with LC and OS rates exceeding 84% and 73% at 24 months post-SRT, respectively.

**Table 2:** LC and OS data from studies using SRT for the management of recurrent STS LC, local control; OS, overall survival; SRT, stereotactic radiotherapy; STS, soft tissue sarcoma

Study	No. of patients	No. of lesions	Location	BED (Gy₁₀)	LC	OS	Toxicity
This study	16	36	Lung, extremities, trunk, spine, abdomen, brain	Median: 96 (range: 48-151)	100% at 12 months; 88% at 18 months	90% at 18 months	One patient with grade 3 skin necrosis
Franceschini et al. (2024) [17]	138	202	Lung, liver, nodes, other	Median: 98.9 (range: 35.7-180)	94.8% at 12 months; 88% at 24 months	91.5% at 12 months; 72.7% at 24 months	Not reported
Loi et al. (2018) [18]	16	26	Lung, soft tissue/lymph nodes	Median: 138 (range: 86-180)	84% at 24 months; 78% at 48 months	73% at 24 months; 54% at 48 months	No toxicity ≥ grade 3
Navarria et al. (2022) [19]	44	71	Lung	≥105	98.5% at 12 months	88.6% at 12 months; 66.7% at 24 months; 48.2% at 60 months	Not reported
Farooqi et al. (2024) [20]	70	98	Lung	≥112	83% at 24 months	87% at 12 months; 65% at 24 months	10% with grade 2 pneumonitis
Baumann et al. (2020) [21]	44	56	Lung	Median: 100 (range: 38.4-112)	96% at 12 months; 90% at 24 months	74% at 12 months; 46% at 24 months	7% with grade 2 chest wall pain; 2% with grade 2 pneumonitis
Dhakal et al. (2012) [22]	14	74	Lung	75	88% at 24 months; 82% at 36 months	Not reported	Not reported
Lindsay et al. (2018) [23]	44	117	Lung	75	95% at 24 months	82% at 24 months; 50% at 60 months	Pneumonitis ≤ grade 3; one patient with esophageal stricture
Zamarud et al. (2023) [25]	23	150	Brain	Median: 60 (range: 42.6-87.5)	30% at 12 months	39% at 12 months	Not reported
Sim et al. (2020) [26]	24	58	Brain	Median: 55 (range: 37.5-81.6)	89% at 12 months	38% at 12 months	Not reported
Flannery et al. (2010) [27]	21	60	Brain	Median: 41.6	88% at four months	61% at 12 months	Not reported

Patients with STS frequently present with pulmonary metastases. Several studies have evaluated the use of SRT for treating these metastases, reporting LC rates exceeding 95% at 12 months and over 83% and 82% at 24 and 36 months post-SRT, respectively [19-23]. The high LC rates are associated with corresponding OS rates ranging from 74% to 91.5% at 12 months, 46% to 72.7% at 24 months, and 48.2% to 50% at 60 months. The LC and OS outcomes reported in the literature are consistent with the results observed in this study.

STS rarely metastasizes in the brain [24]. In our study, one patient with a single metastasis of 2 cm in diameter was treated with a BED of 51 Gy₁₀. Follow-up MRI images showed excellent LC 9 months post-SRT, but the patient was lost on subsequent follow-up. Table 2 summarizes findings from three studies evaluating the efficacy of SRT in STS brain metastases [25-27]. In these studies, median BED values exceeded 41.6 Gy₁₀. Reported LC rates at 12 months post-SRT varied between 30% and 89%, despite the use of comparable BED values. Corresponding OS rates ranged from 38% to 60%. The observed variability in outcomes could be associated with corresponding differences in the total intracranial tumor volume and the extent of the extracranial metastatic burden across the patient cohorts.

SRT treatments are generally associated with minimal toxicities ≥ grade 3. As shown in Table 2, patients treated with SRT for lung metastases have a less than 10% probability of presenting symptomatic pneumonitis (grade 2), which is usually resolved with the use of corticosteroids. Care should be taken for central or ultracentral lesions, where the large bronchus, trachea, and esophagus should be spared from high doses. For these cases, as well as for lung lesions close to the chest wall, a risk-adaptive dose/fractionation approach should be followed involving prescription schemes of 50-55 Gy/4-5 fractions and 60 Gy/8 fractions [19]. Regarding skin toxicity, one patient in this study treated for a local relapse in the upper extremity experienced a severe skin necrosis (grade 3) six months post-treatment. The specific patient had undergone several previous surgeries due to repeated recurrences and reconstruction with skin flap and was treated with a dose/fractionation protocol of 57.6 Gy/12 fractions.

Finally, the toxicity profile of SRT treatments should be compared against corresponding toxicities of other local treatments (e.g., surgery). Regarding surgical toxicity, it is worth noting that a patient in this study was treated using SRT for the local recurrence of the uterine sarcoma, whereas it was referred for surgical resection of its relatively large lung metastasis. The patient died from surgical complications from the operation of the lung metastasis. The efficacy and toxicity of SRT have been compared against surgery in two recent studies [10,11]. Gutkin et al. (2022) analyzed the rates of recurrence, OS, and treatment complications in 217 patients (525 metastatic sarcomas) undergoing either surgical resection or SRT [10]. Local recurrence occurred in 16.5% of the tumors treated with surgery and only in 2.3% of the tumors treated with SRT. The adjusted hazard ratio for local relapse of lesions treated surgically was 11.5 (p = 0.026) when controlling for tumor size and tumor site. Furthermore, a complication rate of 18% was reported for surgery compared to radiation-related toxic effects (none higher than grade 2) of 12% for SRT. Tetta et al. (2020) performed a systematic review to evaluate the outcomes of SRT and metastasectomy (MTS) for the treatment of lung metastases from STS [11]. A total of 1306 patients with STS were analyzed: 1104 underwent MTS and 202 had SRT. The authors showed that local treatment of lung metastases with SRT was associated with a higher survival rate and lower cumulative overall death rate compared to MTS. Both treatment options presented similar OS times and survival rates without disease.

The main limitations of the study include the small sample size and the heterogeneity of the patient cohort, which may limit the generalizability of the findings. Despite these constraints, the results provide valuable insights into the potential role of SRT in treating recurrent sarcoma.

In the absence of randomized trials, clinical practice guidelines for sarcoma treatment with SRT, derived from retrospective series and small prospective phase II studies, may serve as a useful framework [13]. At the same time, reporting real-world data can offer valuable insights into the application of these treatments in routine clinical practice, extending beyond the structured constraints of clinical research protocols.

## Conclusions

The use of SRT for the management of locally recurrent and metastatic STS was evaluated. Excellent LC rates of 26/26 (100%) and 15/17 were found 12 and 18 months post-SRT, respectively. SRT was generally well tolerated, with minimal toxicity observed, except for one case of late skin toxicity in the upper extremity. These findings suggest that SRT is an effective treatment option for selected locally recurrent or metastatic STS patients.
